# Reassortant Group A Rotavirus from Straw-colored Fruit Bat (*Eidolon helvum*)

**DOI:** 10.3201/eid1612.101089

**Published:** 2010-12

**Authors:** Mathew D. Esona, Slavica Mijatovic-Rustempasic, Christina Conrardy, Suxiang Tong, Ivan V. Kuzmin, Bernard Agwanda, Robert F. Breiman, Krisztian Banyai, Michael Niezgoda, Charles E. Rupprecht, Jon R. Gentsch, Michael D. Bowen

**Affiliations:** Author affilations: Centers for Disease Control and Prevention, Atlanta, Georgia, USA (M.D. Esona, S. Mijatovic-Rustempasic, C. Conrardy, S. Tong , I.V. Kuzmin, M. Niezgoda, C.E. Rupprecht, J.R. Gentsch, M.D. Bowen);; National Museum of Kenya, Nairobi, Kenya (B. Agwanda); Centers for Disease Control and Prevention–Kenya, Nairobi (R.F. Breiman);; Hungarian Academy of Sciences, Budapest, Hungary (K. Banyai)

**Keywords:** Straw-colored fruit bat, Eidolon helvum, rotavirus, viruses, reassortment, heterologous genome segments, podcast, zoonoses, research

## Abstract

Bats are known reservoirs of viral zoonoses. We report genetic characterization of a bat rotavirus (Bat/KE4852/07) detected in the feces of a straw-colored fruit bat (*Eidolon helvum*). Six bat rotavirus genes (viral protein [VP] 2, VP6, VP7, nonstructural protein [NSP] 2, NSP3, and NSP5) shared ancestry with other mammalian rotaviruses but were distantly related. The VP4 gene was nearly identical to that of human P[6] rotavirus strains, and the NSP4 gene was closely related to those of previously described mammalian rotaviruses, including human strains. Analysis of partial sequence of the VP1 gene indicated that it was distinct from cognate genes of other rotaviruses. No sequences were obtained for the VP3 and NSP1 genes of the bat rotavirus. This rotavirus was designated G25-P[6]-I15-R8(provisional)-C8-Mx-Ax-N8-T11-E2-H10. Results suggest that several reassortment events have occurred between human, animal, and bat rotaviruses. Several additional rotavirus strains were detected in bats.

## Abstract 2

TOC summary: Bats may be reservoirs of zoonotic viruses that threaten human health.

Rotaviruses are members of the family *Reoviridae* and genus *Rotavirus* and contain 3 primary species: *Rotavirus A*, *Rotavirus B*, and *Rotavirus C* (*1*). The rotavirus genome contains 11 segments of double-stranded RNA encoding 6 structural viral proteins (VP1–VP4, VP6, and VP7) and 6 nonstructural proteins (NSP1–NSP6). Rotavirus A strains are associated with acute infectious diarrhea in humans and animals (*2*). The segmented nature of rotavirus genomes enables reassortment events in which novel rotavirus strains are produced with new combinations of genome segments derived from parental virus strains (*3*). Reassortment is a major mechanism for generating genetic diversity of rotaviruses and driving rotavirus evolution. New rotavirus strains emerge every year as a result of genomic reassortment among cocirculating rotaviruses. Although most rotaviruses appear to be host restricted, interspecies transmission of rotaviruses has been documented (*4*–*8*). Recently, new genotypes found in a variety of animals have been reported (*9*). Thus, monitoring rotaviruses in domesticated and wild animals can potentially identify emerging human and veterinary pathogens.

Species A rotaviruses have been traditionally classified by using a binomial nomenclature based on serotype and genotype specificities of the outer capsid antigens, VP7 (G-type) and VP4 (P-type). Recently, Matthijnssens et al. (*10*) proposed a classification system based on all 11 genome segments (*4*,*7*,*10*). This scheme uses specific nucleotide sequence identity cutoff values for the complete open reading frame (ORF) of each gene segment to delineate genotypes, and new genotypes are formally assigned by the Rotavirus Classification Working Group (RCWG) (*10*).

Bats of many species are being recognized as reservoir hosts for viruses that can cross species barriers to infect humans (*11*). Such viruses include Ebola and Marburg viruses, Nipah and Hendra viruses, severe acute respiratory syndrome–like coronavirus, rabies and other lyssaviruses, togaviruses, flaviviruses, bunyaviruses, and members of the family *Reoviridae* (*11*–*13*). The straw-colored fruit bat (*Eidolon helvum*, family Pteropodidae, order Chiroptera) (*11*) is widely distributed and ranges from the southwestern Arabian Peninsula to the forest and savanna zones (south of the Sahara Desert) and offshore islands of Africa. Detection of Lagos bat virus (*Lyssavirus*) (*14*) and Ife virus (*Orbivirus*) (*15*) in *E. helvum* bats demonstrates their potential as reservoirs of viruses that cause zoonotic diseases. Rotaviruses have not been detected in bats, although other viruses in the family *Reoviridae* have been detected in bats (*11*).

The purpose of this study was to examine *E. helvum* bats for rotaviruses and characterize any strains found. We detected a novel rotavirus A species in *E. helvum* bats. This isolate contained a VP4 gene that probably originated from a human rotavirus, an NSP4 gene of likely human or animal rotavirus origin, and otherwise a unique genetic background requiring establishment of new genotypes for at least 7 genes.

## Materials and Methods

### Samples

During 2007, fecal swabs were collected from bats during field surveys conducted in Kenya as described (*14*). Samples tested for rotaviruses were first screened for lyssaviruses and coronaviruses (*13*).

### Nucleic Acid Extraction and Reverse Transcription–PCR

Suspensions of fecal swabs obtained from *E. helvum* bats were prepared in phosphate-buffered saline. Total nucleic acid was extracted by using the QIAamp Mini Viral Elute Kit (QIAGEN, Valencia, CA, USA). After denaturing extracted nucleic acid at 95°C for 5 min, reverse transcription–PCR (RT-PCR) for amplification of the rotavirus VP6 gene was performed by using a One-Step RT-PCR Kit (QIAGEN). VP6F and VP6R primers and cycling conditions have been described (*16*). For VP6-positive samples, attempts were made to amplify different rotavirus gene segments (complete or partial) by using published rotavirus-specific consensus primers for VP4 and VP7 (*17*–*19*) and VP1, VP2, VP6, NSP1, NSP2, NSP3, NSP4, and NSP5 (*5*). New internal oligonucleotide primers for VP1, VP2, VP7, NSP2, NSP3, NSP5, and VP6 genes were designed on the basis of initial bat sequences obtained and sequences of published mammalian and avian rotavirus strains and used for sequencing (Table). RT-PCR products were analyzed by performing electrophoresis on 1% agarose gels containing GelRed Gel Stain (Biotium, Inc., Hayward, CA, USA) and viewed by using UV transillumination.

**Table:** Newly designed primers used for amplification and sequencing of bat rotavirus genes*

Primer	Sequence, 5′ → 3′	Gene	Nucleotide position, strand
VP1_Bat	GCT TCG AAT GGA GAA TCG CG	VP1	596–576, –
VP1_Bat	CTC CTG GTG TGT ACC TAC C	VP1	485–504, +
VP1_Bat	CCT CGT GTG TAA ATA CGG ACA	VP1	1130–1151, +
VP2_Bat	CAG AGC AGG CTA AGA AGC AGA CTA	VP2	393–370, –
VP2_Bat	GCA GCA CCA ATT TGG GTT GAG	VP2	810–830, +
VP2_Bat	CTC AAC CCA AAT TGG TGC TG	VP2	830–810, –
VP2_Bat	CTG AAT CTG AGC TTC AGT TG	VP2	1206–1187, –
VP2_Bat	GAT GTA GCT AGA GTG CCA G	VP2	2247–2265, +
VP2_Bat	CTG GCA CTC TAG CTA CAT C	VP2	2265–2247, –
VP2_Bat	GGT GGC GAA TTA TGA TTG G	VP2	2723–2741, +
VP6_Bat	TCG GTC TAT GGA ATG TGA AAC CTG TTC	VP6	380–354, –
VP7_Bat	CAG ATG TCG TCG ATA ATG	VP7	693–710, +
VP7_Bat	TGG CGT ACG CAG TGT CCA TTG AC	VP7	218–196, –
VP7_Bat	CGA GTT GGC GTA CGC AGT G	VP7	258–240, –
NSP2_Bat	CAA CTT CCA CAT TGT AAA CGC	NSP2	574–555, –
NSP2_Bat	CTT TCG CGA ATA CAC TGG T	NSP2	1087–1068, –
NSP2_Bat	CT GAT AGA GTG TAT GCG AC	NSP2	727–747, +
NSP3_Bat	CCT CAC TCT CAT CTT TCG GGT CTT C	NSP3	401–376, –
NSP3_Bat	GGA GTT ACC GAG TGA AGC GAA GG	NSP3	673–695, +
NSP5_Bat	ACG CCA GCA TCT GCA TTT GTC	NSP5	279–258, –

*VP, viral protein; NSP, nonstructural protein.

### Nucleotide Sequencing

Specific RT-PCR amplicons were excised from agarose gels and purified by using the QIAquick Gel Extraction Kit (QIAGEN). Cycle sequencing of each amplicon was performed with the same consensus primers used for RT-PCR by using a Big Dye Terminator 1.1 or 3.1 Cycle Sequencing Ready Reaction Kit (Applied Biosystems, Foster City, CA, USA). Cycle sequencing products were purified by using a BigDye XTerminator Purification Kit (Applied Biosystems) or an in-house magnetic bead purification method (S. Mijatovic-Rustempasic et al., unpub. data). Sequencing was performed by using an ABI 3130XL Sequencer (Applied Biosystems). Primer walking sequencing was performed to cover each gene segment on both strands.

### Nucleotide and Protein Sequence Analysis

Sequence chromatogram files were edited and sequence contigs were assembled by using Sequencher 4.8 software (Gene Codes Corporation, Inc., Ann Arbor, MI, USA). Nucleotide sequences were translated into amino acid sequences and manually aligned by using GeneDoc software (*20*). Nucleotide and protein similarity searches were performed by using BLAST (http://blast.ncbi.nlm.nih.gov/Blast.cgi) to search GenBank databases (*21*). Nucleotide and amino acid identities were calculated by using the *P*-distance algorithm of MEGA 4.0 software (*22*). Multiple sequence alignments were performed by using GeneDoc software (*20*). Phylogenetic relationships were inferred from aligned nucleotide sequences and amino acid sequences (VP1 and VP7 proteins only) by Bayesian analysis in MrBayes 3.1.2 (*23*,*24*). Each nucleotide sequence analysis was run for 500,000–700,000 generations until the average SD of split frequencies was <0.01. Bayesian analysis of amino acid data was run for 100,000 generations, after which the average SD of split frequencies was <0.05. Tree files were visualized by using TreeDyn 198.3 (*25*).

## Results

**Figure 1:**
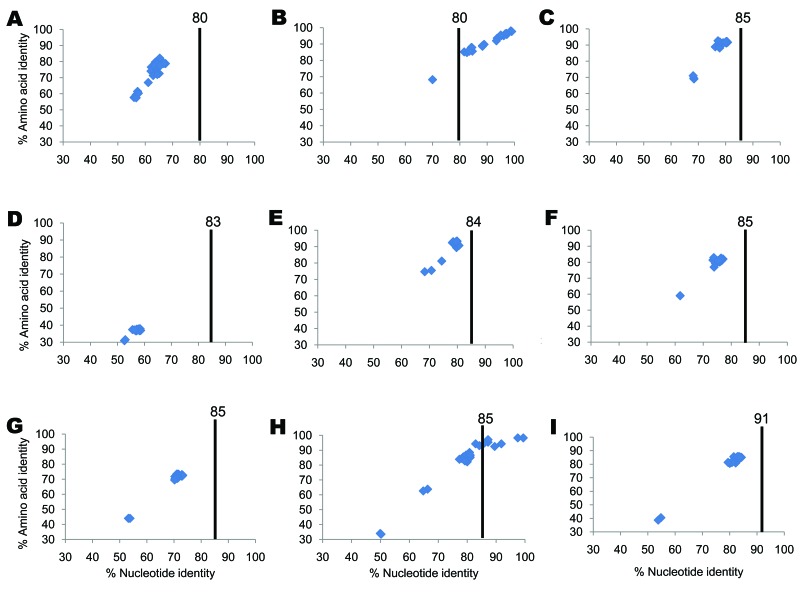
Percentage nucleotide and deduced amino acid homologies of A) viral protein 7 (VP7), B) VP4, C) VP6, D) VP1, E) VP2, F) nonstructural protein 2 (NSP2), G) NSP3, H) NSP4, and I) NSP5 gene segments of bat rotavirus strain Bat/KE4852/07 from Kenya compared with respective genes deposited in GenBank. Vertical lines indicate nucleotide percentage identity cutoff values defining genotypes for 11 rotavirus gene segments (7,10). Blue diamonds indicate coordinates for each pairwise comparison when percentage nucleotide identity is plotted against percentage amino acid identity. GenBank accession numbers used in this comparison are listed in the Technical Appendix.

We initially screened 6 samples; 1 sample, Bat/KE4852/07, obtained from an *E. helvum* bat trapped in Vihiga, Kenya, was positive for rotavirus by VP6 RT-PCR. Using primers annealing to noncoding regions of each segment and internal primers, we then obtained full-length ORF sequences for VP2, VP6, VP7, NSP2, NSP3, NSP4, and NSP5, except for VP1 and VP4, for which partial-length gene sequences were obtained (for the remainder of this report, we will refer to each ORF, from ATG to stop codon, as a gene). None of the sequences reported in this study were inferred from primer sequences. Sequences were not obtained for VP3 and NSP1, despite repeated attempts to obtain amplicons by using panels of rotavirus A–specific primer pairs. Nucleotide sequences for VP1, VP2, VP4, VP6, VP7, NSP2, NSP3, NSP4, and NSP5 were deposited in GenBank under accession nos. GU983672–GU983680. Genetic analyses of Bat/KE4852/07 indicated that 7 genes were unique and 2 were similar to described rotavirus genotypes. Results are summarized below and in Figure 1.

### VP7 Gene

The putative VP7 gene of strain Bat/KE4852/07 was 981 bp and encoded a 326 aa protein. The nucleotide sequence of Bat/KE4852/07 VP7 showed low levels of identity to the 24 established G genotypes (range 55.9%–67.4%) (Figure 1, panel A). The VP7 gene of Bat/KE4852/07 was classified into a novel VP7 genotype, G25, by the RCWG (*10*). When compared with other mammalian rotavirus VP7 protein sequences, amino acid dissimilarity was >17% overall (Figure 1, panel A) and exceeded 20% in antigenic regions A, B, and C (*26*).

### VP4 Gene

An 829-bp region of the VP4 gene of Bat/KE4852/07 strain was amplified and sequenced by using standard VP4 RT-PCR primers (*18*). The sequence corresponded to bases 24–851 of strain US1205 VP4 (GenBank accession no. AF079356.) BLAST searches indicated that the bat rotavirus VP4 sequence was closely related to human P[6] strains from Africa (6809/ARN) and eastern Asia (CAU214) and showed 99% nt and 98% aa identities (Figure 1, panel B). Relatively low sequence identities were found when compared with P[6] sequences of animal origin (81.6% nt and 85.2% aa identities) (Figure 1, panel B).

### VP6 Gene

The complete VP6 gene (1,194 bp), with a protein of 397 aa, of Bat/KE4852/07 strain was determined. Overall nucleotide identity with reference genotypes I1-I13 strains ranged from 68.1% to 80.6% (Figure 1, panel C). These nucleotide identity values fell below the VP6 genotype cutoff value of 85% (*10*), indicating that Bat/KE4852/07 strain belongs to a novel VP6 genotype designated I15 by the RCWG. Bat/KE4852/07 VP6 amino acid sequence shared 69%–92.7% identity with other rotavirus VP6 sequences (Figure 1, panel C).

### VP1 Gene

A fragment of the VP1 gene was obtained for Bat/KE4852/07. The sequenced region was 1,198 nt, which was one third the expected full length of the VP1 gene and corresponded to bases 252–1451 of rotavirus strain S2 VP1 sequence (GenBank accession no. DQ870485). Comparison of the partial VP1 nucleotide and amino acid sequences of Bat/KE4852/07 with those of other mammalian and avian rotavirus strains showed low levels of identity similar to those of other VP1 genotypes (<59% and <38%, respectively; Figure 1, panel D).

**Figure 2:**
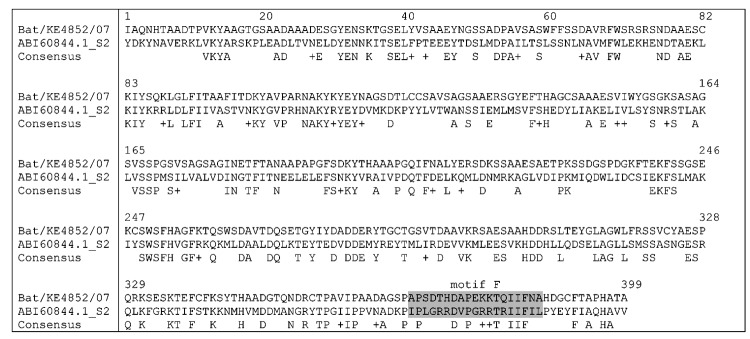
Alignment of viral protein 1 (VP1) amino acid sequence of bat rotavirus strain KE4852/07 from Kenya with cognate VP1 sequence of reference rotavirus A strain S2. The consensus line shows conserved amino acid residues and similar residues (indicated by +). The motif F region (*27*) is shaded.

Despite the uniqueness of the bat rotavirus VP1 sequence, BLAST search and alignment results with amino acid sequence displayed the highest degree of identity with rotavirus A VP1 sequences (48% conservation of similar amino acid residues) (Figure 2). The Bat/KE4852/07 VP1 gene partial sequence includes the region encoding the 19 residue polymerase F motif described (*27*), but within this domain, the percent amino acid identity with other group A rotaviruses is <37% (53% similarity), and none of the 3 arginine residues (RR452, R457, R460) of a predicted functional role in rotaviruses are present in Bat/KE4852/07 VP1 (Figure 2). The partial gene nucleotide sequence identity was well below the cutoff value of 83% that has been used to classify VP1 genotypes. The amino acid sequence divergence (>62%) exceeds that observed when group A and C rotavirus VP1 proteins are compared (>53%) (*27*). Although the minimal sequence length set by the RCWG for new candidate genotypes does not permit official designation of a new genotype derived from the partial gene sequence of Bat/KE4852/07, we showed that this bat rotavirus contains a highly divergent VP1 gene provisionally designated genotype R8.

### VP2 Gene

The VP2 gene of strain Bat/KE4852/07 was 2,712 bp and encoded a deduced protein of 903 aa. The sequence of the VP2 gene segment of Bat/KE4852/07 strain was longer than those of most rotavirus strains because of several nucleotide insertions within the ORF near the 5′ end of the gene. As a result of these insertions, the predicted VP2 protein was 21 aa longer than that of most mammalian and avian rotaviruses. Nucleotide sequence comparisons indicated that the VP2 sequence of Bat/KE4852/07 was distantly related to all 6 established VP2 genotypes; identities ranged from 68.3% to 80.6% (Figure 1, panel E). These values were below the cutoff value of 84% nt sequence identity that has been used to classify VP2 genotypes (*10*). Thus, Bat/KE4852/07 was assigned into novel genotype C8 by the RCWG. VP2 amino acid identities between Bat/KE4852/07 and other rotaviruses were <94% (Figure 1, panel E).

### NSP2 Gene

The NSP2 gene of strain Bat/KE4852/07 was 954 bp and encoded a polypeptide of 317 aa. Bat/KE4852/07 NSP2 gene sequence shared <78% identity with other rotavirus NSP2 strains (Figure 1, panel F). This value was below the cutoff value of 85% nt sequence identity that has been used to classify NSP2 genotypes (*10*). The RCWG assigned novel genotype N8 to the NSP2 gene of strain Bat/KE4852/07.

### NSP3 Gene

The NSP3 gene of strain Bat/KE4852/07 was 936 bp and encoded a deduced protein of 311 aa. Bat/KE4852/07 strain NSP3 nucleotide and amino acid sequences exhibited <74% identity with other NSP3 genotypes (Figure 1, panel G). Because this value is below the cutoff value of 85% nt identity used to differentiate T genotypes (*10*), this gene was assigned to a novel NSP3 genotype (T11) by the RCWG.

### NSP4 Gene

The putative NSP4 gene was 528 bp and encoded a polypeptide of 175 aa. Analyses of nucleotide and deduced amino acid sequences indicated that the Bat/KE4852/07 NSP4 gene shared >99% nt and >98% aa identities with human (I321, B1711 and DS-1), ovine (OVR762), simian (PTRV), and bovine (WC3) rotavirus strains of the NSP4 genotype E2 (Figure 1, panel H).

### NSP5 Gene

The NSP5 gene of strain Bat/KE4852/07 was 630 bp and encoded a 209-aa polypeptide. This gene was longer than NSP5 genes of most rotavirus strains because of several nucleotide insertions within the ORF. Thus, the putative NSP5 is 11 aa longer than that of most mammalian and avian rotaviruses. The NSP5 gene of strain Bat/KE4852/07 shared <85% nt identity with other NSP5 genotypes (*10*) (Figure 1, panel I) and was assigned to a novel NSP5 genotype (H10). Within the NSP5 gene of strain Bat/KE4852/07, there was a second ORF at nucleotide positions 80–346 (+1 reading frame) which corresponded to a putative NSP6 gene.

### Phylogenetic Relationships of Bat/KE/4852/07 Genes to other Rotavirus Strains

**Figure 3:**
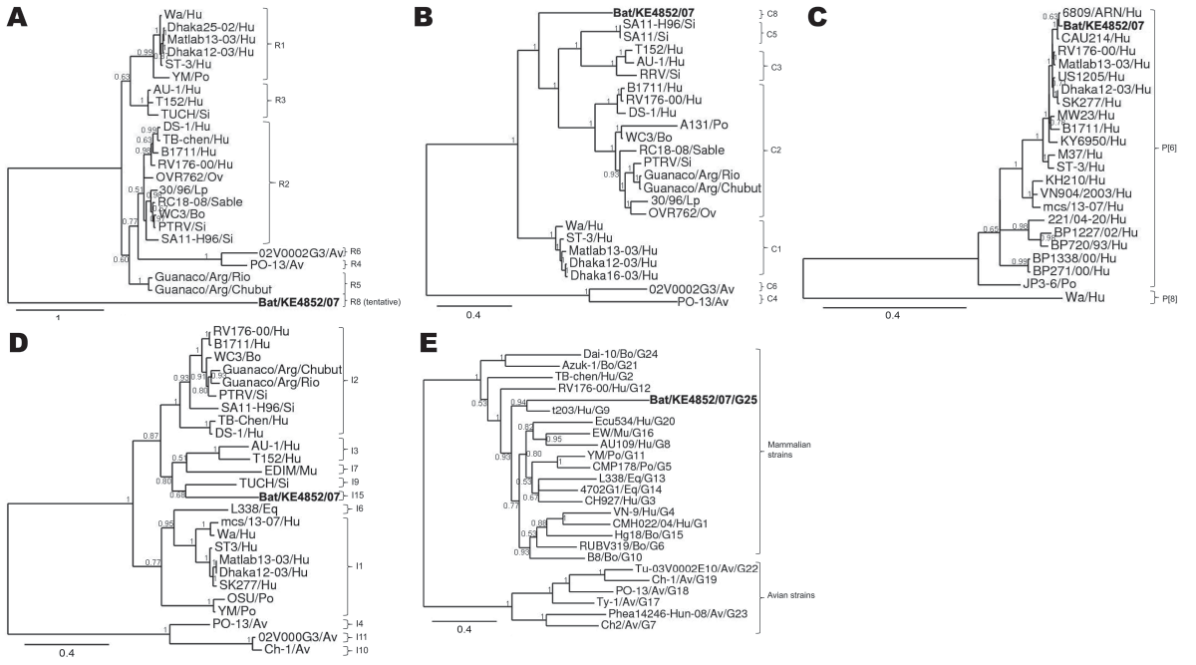
Phylograms indicating genetic relationships of partial or complete nucleotide sequences of A) viral protein 1 (VP1) partial, B) VP2, C) VP4 partial, D) VP6, and E) VP7 of bat rotavirus strain Bat/KE4852/07 (**boldface**) from Kenya with representatives of known human and animal rotavirus genotypes. Posterior probability values are indicated at each branch node. Scale bars indicate nucleotide substitutions per site. GenBank accession numbers of all strains used are listed in the Technical Appendix. Genotypes of each gene segment characterized in this study are listed to the right of each tree. Hu, human; Po, porcine; Si, simian; Ov, ovine; Lp, lapine; Bo, bovine; Av, avian; Eq, equine.

**Figure 4:**
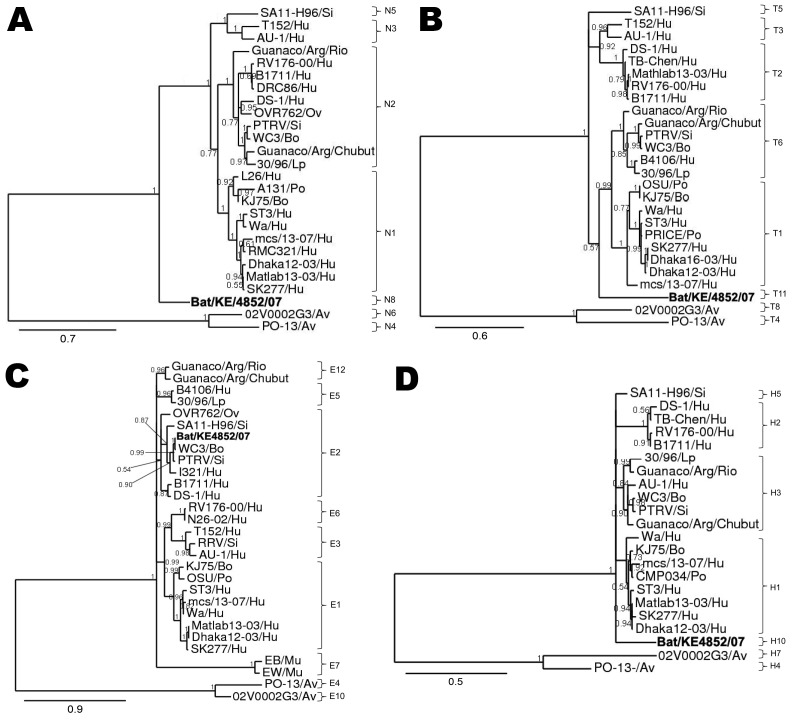
Phylograms indicating genetic relationships of partial or complete nucleotide sequences of A) nonstructural protein 2 (NSP2), B) NSP3, C) NSP4, and D) NSP5 of bat rotavirus strain Bat/KE4852/07 (**boldface**) from Kenya with representatives of known human and animal rotavirus genotypes. Posterior probability values are indicated at each branch node. Scale bars indicate nucleotide substitutions per site. GenBank accession numbers of all strains used are listed in the Technical Appendix. Genotypes of each gene segment characterized in this study are listed to the right of each tree. Si, simian; Hu, human; Ov, ovine; Bo, bovine; Lp, lapine; PO, porcine; Av, avian; Mu, murine.

Bayesian phylogenetic analyses of Bat/KE4852/07 and other rotavirus strain sequences indicated that 2 genes (VP4 and NSP4) were closely related to described mammalian rotaviruses; that 6 bat rotavirus genes (VP2, VP6, VP7, NSP2, NSP3, NSP5) were more distantly related and represented novel genotypes; and that the VP1 gene was distant from all known cognate genes of mammalian and avian rotaviruses (Figures 3, 4). In the phylogeny estimated from VP7 nucleotide sequences, Bat/KE4852/07 occupied a well-supported (posterior probability 0.94) terminal group with G9 rotavirus strain t203 (AY003871), although the bat strain was connected by a long branch to the terminal node (Figure 3, panel E). Bayesian analysis of VP7 amino acid sequences yielded the same result.

The VP6 phylogenetic estimate grouped Bat/KE4852/07 with the I9 genotype TUCH strain (EF583013) (posterior probability 0.81) and a long terminal branch (Figure 3, panel D). Phylogenetic analysis of VP2 sequences resulted in well-resolved phylogeny with Bat/KE4852/07 and occupied an intermediate lineage between C1 genotype rotaviruses and a clade containing C2, C3, and C5 viruses (Figure 3, panel B). Analysis of NSP3 sequences indicated weak support (posterior probability 0.57) for monophyly of the bat rotavirus with T1 and T6 genotype viruses, and Bat/KE4852/07 was separated from these other mammalian viruses by a relatively long terminal branch (Figure 4, panel B). Bayesian analysis of NSP5 sequences indicated strong support (posterior probability 1.0) for monophyly of the bat rotavirus with mammalian rotaviruses in genogroups H1, H2, H3, and H5. However, intergenogroup relationships within this clade were not resolved (Figure 4, panel D).

Bayesian analysis of partial VP4 sequences yielded a phylogenetic estimate that places the bat rotavirus strain within a clade containing all genotype P[6] viruses (posterior probability 1.0) and occupying a lineage shared with human P[6] strains 6809/ARN and CAU214 (Figure 3, panel C). The phylogenetic estimate obtained by using NSP4 gene nucleotide data supported the monophyletic origin of Bat/KE4852/07 with human strain I321 (AF165066 genotype E2), bovine strain WC3 (AY050273, genotype E2), and simian strain PTRV (FJ422140, genotype E2) (Figure 4, panel C).

For partial VP1 sequences, Bayesian analyses of nucleotide and amino acid data did not produce well-resolved phylogenies. However, in each tree the longest terminal branch was between the bat rotavirus and the ancestral node (Figure 3, panel A). Genetic distance separating Bat/KE4852/07 from the other rotaviruses was longer than the distance between avian rotaviruses (genotypes R4 and R6) and mammalian rotaviruses. For NSP2, the bat rotavirus gene occupied an intermediate position between avian rotaviruses (genotypes N4 and N6) and mammalian rotaviruses (genotypes N1, N2, N3, and N5) and showed maximum posterior probability support (Figure 4, panel A).

### Additional Screening Results

After characterization of Bat/KE4852/07, we processed an additional 39 *E. helvum* bat fecal swab samples from Kenya and screened them for rotaviruses by using VP6 RT-PCR. Three additional samples (Bat/KE5096/07, Bat/KE5105/07, and Bat/K5175/07) were positive for rotavirus. These 3 samples were obtained in Maseno, Kenya, which is ≈20 km from Vihiga. Given the population dynamics and migratory patterns of this species, bats from both roosts likely interact, at least during certain times of the year. VP6 sequences for Bat/KE5096/07, Bat/KE5105/07, and Bat/K5175/07 samples were 100% identical to VP6 sequence of Bat/KE4852/07. In an attempt to obtain a complete genomic sequence for the bat rotavirus, we will analyze this virus by using sequence-independent deep sequencing.

## Discussion

We detected and genetically characterized a bat-associated rotavirus strain. Although NSP1 and VP3 gene sequences remain undefined, this incomplete genome sequence provides insight into rotavirus diversity, evolution, classification, and ecology. The RCWG has classified bat strain Bat/KE4852/07 as G25-P[6]-I15-R8(provisional)-C8-Mx-Ax-N8-T11-E2-H10. The finding that the Bat/KE4852/07 VP4 gene was nearly identical to human P[6] strains suggests that it has been introduced as a result of a ressortment event between human and bat rotaviruses. The NSP4 gene of this bat rotavirus is also likely to have been introduced into the bat rotavirus genome by reassortment with human or animal strains. The possibility that the VP4 and NSP4 gene segments were originally bat rotavirus genes, which have evolved and become ubiquitous among human rotaviruses for several years, cannot be excluded. However, this possibility is less likely because 7 of the Bat/KE4852/07 genes are genetically divergent from any previously reported rotavirus genotype for humans or animals.

This study provides evidence that human rotavirus gene segments can reassort naturally into an animal rotavirus backbone. Reassortment of animal rotavirus segments onto human strain backbones has been documented (*28*), and experimental insertion of human rotavirus strains onto animal strain backbones was the basis for construction of reassortant rotavirus vaccines Rotashield and RotaTeq (*29*). Detection of human rotavirus strain components in domestic animals has been reported (*30*,*31*) but it is not known whether these strains were reassortant viruses or human strains that infected animals because only G and P types of these viruses were reported. Documentation of a human-to-bat transmission event of any infectious virus is unprecedented, and the genetic bases of such interactions deserve additional studies (*8*).

Human and fruit bat activities provide ample opportunities for *E. helvum* bats and humans to contact each other and their respective rotaviruses. Fruit bats often live near human habitats (*11*) as a result of deforestation and expanded settlement of human populations. Fruit bats must forage for food over wide areas and often feed in orchards. A proposed mechanism for bat infection with human rotaviruses would be through use of night soil (human feces) as fertilizer on farmlands and orchards, a common practice throughout Africa and Asia (*32*,*33*). Night soil contains pathogenic bacteria, viruses, and parasites linked with disease endemicity in these countries (*32*–*34*). Human rotaviruses have been detected in surface water, reservoirs, and sewage; and viable virus has been isolated from drinking water (*35*,*36*). *E. helvum* bats have has been observed skimming bodies of water in Africa, presumably to collect water for drinking (*37*), and surface water could be contaminated with viable human rotaviruses by night soil fertilizer or inadequate sanitation practices. Contact with human feces during drinking or feeding provides a mechanism by which fruit bats can ingest human rotaviruses, which would serve as the source of heterologous rotavirus genes acquired by reassortment. Additional information is needed about the actual mechanisms and frequency of rotavirus transmission between humans and bats.

The partial VP1 gene sequence obtained for Bat/KE4852/07 is more divergent than other group A rotavirus VP1 genes and does not contain several conserved motifs in VP1 proteins of group A, B, and C rotaviruses (*27*). The level of divergence suggests that the origin of this gene may be outside group A rotaviruses, perhaps from a yet-to-be-defined rotavirus group distinct from groups A, B, C, and the ADRV-N/J19 group (*38*). Group A and C rotaviruses can replicate each other’s plus-strand RNAs, and it has been speculated that rotaviruses from these 2 groups can exchange genomic segments if they co-infect the same cell (*27*), although this phenomenon has not been demonstrated empirically. We propose that the Bat/KE4852/07 VP1 gene (segment 1) originated from a nonspecies A rotavirus, most likely a currently undefined or unrecognized rotavirus group.

Each year, group A rotaviruses cause ≈600,000 deaths among children worldwide (*39*). Current surveillance studies are focused mainly on humans, and to a lesser extent, on domesticated animals and pets. Recent studies have identified new rotavirus genotypes in many captive and free-ranging wildlife (*9*). However, relatively little is known about the prevalence of rotaviruses among wildlife. Results of our study reinforce previous initiatives to enhance animal rotavirus surveillance, including corresponding information about wildlife rotaviruses, not only to investigate the genetic variability of wildlife rotaviruses, but also to understand the origin of unusual rotavirus strains in various hosts, including humans, especially within the One World, One Health concept described by the Wildlife Conservation Society (*40*).

In summary, although a limted amount of stool sample resulted in our inability to complete the full genome of Bat/KE4852/07, discovery of a rotavirus in bats and genetic analysis of this virus provide new insight into the ecology and evolution of rotaviruses. Our findings not only reinforce the potential role of bats as reservoirs of zoonotic viruses that are threats to human health (*11*), but also suggest that humans can serve as reservoirs of virus, which can result in anthropozoonotic transmission of rotavirus genes. Global surveillance for rotaviruses in bats and other wildlife is needed to corroborate, elaborate, and more fully understand the origin, adaptation, and evolution of bat rotavirus strains. In addition, further research is needed to establish the precise role of bats in the natural history of this rotavirus.

## Supplementary Material

Technical Appendix GenBank accession numbers of strains used in sequence and phylogenetic analyses of rotaviruses.
