# Supplementary material for: Reassortant Group A Rotavirus from Straw-colored Fruit Bat (Eidolon helvum)
Source: Emerg Infect Dis. 2010 Dec;16(12):1844–52. doi: 10.3201/eid1612.101089 (PMC3294550; doi:10.3201/eid1612.101089)
Supplement: Technical Appendix — GenBank accession numbers of strains used in sequence and phylogenetic analyses of rotaviruses. [file 10-1089-Techapp_4p.pdf]

Article DOI: 10.3201/eid1612.101089

Reassortant Group A Rotavirus from Straw-colored Fruit Bat (*Eidolon helvum*)

Technical Appendix

GenBank accession numbers of strains used in sequence and phylogenetic analyses of rotaviruses. VP, viral protein; Hu, human; Bo, bovine; Av, avian; Lp, lapine; Si, simian; Ov, ovine; Po, porcine; Eq, equine; Mu, murine; NSP, nonstructural protein.

VP1

AY787653 (TB-Chen/Hu/R2), EF560615 (WC3/Bo/R2), DQ146699 (T152/Hu/R3), AB009629 (PO-13/Av/R4), FJ169853 (02V0002G3/Av/R6), DQ146649 (Dhaka25–2/Hu/R1), DQ205221 (30/96/Lp/R2), FJ347122 (Guanaco/Arg/Rio Negro/1998/R5), FJ347100 (Guanaco/Arg/Chubut/1999/R5), DQ838640 (SA11 [H96]/Si/R2), DQ490539 (Wa/Hu/R1), DQ490533 (AU-1/Hu/R3), DQ870505 (DS-1/Hu/R2), EF554148 (OVR762/Ov/R2), DQ146660 (Dhaka12–03/Hu/R1), EF583045 (ST-3/Hu/R1), FJ495126 (RC 18/08/Sable/R2), FJ422131 (PTRV/Si/R2), DQ146671 (Matlab13–03/Hu/R1), DQ490551 (RV176–00/Hu/R2), EF554082 (B1711/Hu/R2) and EF583010 (TUCH/Si/R3).

VP2

EF560616 (WC3/Bo/C2), DQ490552 (RV176–00/Hu/C2), DQ146700 (T152/Hu/C3), AB009630 (PO-13/Av/C4), X16831 (SA11/Si/C5), FJ169854 (02V0002G3/Av/C6), DQ492670 (Dhaka16–03/Hu/C1), DQ205222 (30/96/Lp/C2), DQ838635 (SA11 [H96]/Si/C5), EF554149 (OVR762/Ov/C2), EF560619 (A131/Po/C2), FJ495127 (RC 18/08/Sable/C2), DQ870506 (DS-1/Hu/C2), DQ490536 (AU-1/Hu/C3), X14942 (Wa/Hu/C1), FJ347123 (Guanaco/Arg/Rio Negro/1998/C2), FJ347101 (Guanaco/Arg/Chubut/1999/C2), DQ146661 (Dhaka12–03/Hu/C1), EF583046 (ST-3/Hu/C1), FJ422132 (PTRV/Si/C2), DQ146672 (Matlab13–03/Hu/C1), EF554083 (B1711/Hu/C2), DQ492670 (Dhaka16–03/Hu/C1), and EF583007 (RRV/Si/C3).

VP4

FJ425172 (6809/2000/ARN/Hu/P6), EF059921(CAU 214/Hu/P6), DQ490554 (RV176–00/Hu/P6), DQ146674 (Matlab13–03/Hu/P6), AF079356 (US1205/Hu/P6), DQ146663 (Dhaka12–03/Hu/P6), EU839948 (SK277/Hu/P6), AJ278253 (MW23/Hu/P6), EF554085 (B1711/Hu/P6), FJ386450 (KY6950/Hu/P6), L20877 (M37/Hu/P6), EF672612 (ST-3/Hu/P6), AB271687 (KH210/Hu/P6), EF179118 (VN904/2003/Hu/P6), EU753965 (mcs/13–07/Hu/P6), AY955303 (221/04–20/Hu/P6), AJ621505 (BP1227/02/Hu/P6), AJ621507 (BP1338/00/Hu/P6), AJ621502 (BP271/00/Hu/P6), AJ621503 (BP720/93/Hu/P6), AB176685 (JP3–6/Hu/P6), and L34161 (Wa/Hu/P8).

VP6

AY787645 (TB-Chen/Hu/I2), AF411322 (WC3/Bo/I2), DQ490555 (RV176–0/Hu/I2), AY594670 (TUCH/SiI9), U65988 (EDIM/Mu/I7), DQ146702 (T152/Hu/I3), AF317123 (OSU/Po/I1), X69487 (YM/Po/I1), DQ146664 (Dhaka12–03/Hu/I1), D82974 (L338/Eq/I6), D16329 (PO-13/Av/I4), DQ490538 (AU-1/Hu/I3), DQ870507 (DS-1/Hu/I2), K02086 (Wa/Hu/I1), FJ347126 (Guanaco/Arg/Rio Negro/1998/I2), FJ347104 (Guanaco/Arg/Chubut/1999/I2), EF583048 (ST-3/Hu/I1), FJ422136 (PTRV/Si/I2), FJ169858 (02V0002G3/Av/I11), EU839971 (SK277/Hu/I1), DQ146675 (Matlab13–03/Hu/I1), EF554086 (B1711/Hu/I2), EU753964 (mcs/13–07/Hu/I1), and D82970 (Ch-1/Av/I10).

VP7

EF199717 (CMH022/04/Hu/G1), FJ598040 (VN-9/Hu/G4), D86275 (CH927/Hu/G3), AY750923(4702G1/Eq/G14), DQ515961(CMP178/Po/G5), M23194(YM/Po/G11), AY003871 (t203/Hu/G9), EF199501(RUBV319/Bo/G6), AB272753 (AU109/Hu/G8), U08430 (EW/Mu/G16), D13549 (L338/Eq/G13), DQ490556 (RV176–00/Hu/G12), AF386920 (B8/Bo/G10), EU805775 (Ecu534/Hu/G20), AY787646 (TB-chen/Hu/G2), AF237666 (Hg18/Bo/G15), AB454421 (Azuk-1/Bo/G21), AB513837 (Dai-10/Bo/G24), X56784 (Ch-2/Av/G7), FN393054 (Phea14246-Hun-08/Av/G23), L01098 (Ty-1/Av/G17), D82979 (PO-13/Av/G18), AB080738 (Ch-1/Av/G19), and EU486973 (Tu-03V0002E10/Av/G22).

NSP2

DQ838615 (SA11-H96/Si/N5), DQ146703 (T152/Hu/N3), DQ490534 (AU-1/Hu/N3), DQ005118 (DRC86/Hu/N2), EF672580 (DS-1/Hu/N2), EF554155 (OVR762/Ov/N2), EF990700

(WC3/Bo/N2), DQ205227 (30/96/Lp/N2), EF672594 (L26/Hu/N1), EF990688 (A131/Po/N1), DQ494402 (KJ75/Bo/N1), EF672615 (ST-3/Hu/N1), L04534 (Wa/Hu/N1), AF506293 (RMC321/Hu/N1), FJ347129 (Guanaco/Arg/Rio Negro/1998/N2), FJ347107(Guanaco/Arg/Chubut/1999/N2), FJ422139 (PTRV/Si/N2), FJ169860 (02V0002G3/Av/N6), EU839973 (SK277/Hu/N1), DQ146678 (Matlab13–03/Hu/N1), DQ490558 (RV176–00/Hu/N2), EF554089 (B1711/Hu/N2), AB009625 (PO-13/Av/N4), EU753970 (mcs/13–07/Hu/N1) and DQ146667 (Dhaka12–03/Hu/N1).

NSP3

AY787649 (TB-Chen/Hu/T2), X81434 (Wa/Hu/T1), EF672579 (DS-1/Hu/T2), DQ146704 (T152/Hu/T3), X81431 (OSU/Po/T1), EF672614 (ST-3/Hu/T1), DQ146668 (Dhaka12–03/Hu/T1), AB009626 (PO-13/Av/T4), FJ169859 (02V0002G3/Av/T8), DQ490535 (AU-1/Hu/T3), DQ205228 (30/96/Lp/T6), DQ838610 (SA11 [H96]/Si/T5), DQ494404 (KJ75/Bo/T1), X81432 (PRICE/Po/T1), DQ146679 (Matlab13–03/Hu/T2), AY740733 (B4106/Hu/T6), FJ347130 (Guanaco/Arg/Rio Negro/1998/T6), FJ347108(Guanaco/Arg/Chubut/1999/T6), FJ422137 (PTRV/Si/T6), EU839974 (SK277/Hu/T1), DQ490559 (RV176–00/Hu/T2), EF554090 (B1711/Hu/T2), DQ492677 (Dhaka16–03/Hu/T1), EF990701 (WC3/Bo/T6), and EU753969 (mcs/13–07/Hu/T1).

NSP4

DQ490560 (RV176–00/Hu/E6), U96335(EW/Mu/E7), DQ146705 (T152/Hu/E3), D88831 (OSU/Po/E1), U59110 (ST-3/Hu/E1), DQ146669 (Dhaka12–03/Hu/E1), AB009627 (PO-13/Av/E4), FJ169862 (02V0002G3/Av/E10), DQ205230 (30/96/Lp/E5), AY740732 (B4106/Hu/E5), DQ494398 (KJ75/Bo/E1), AB112917 (EB/Mu/E7), DQ146691 (N26–02/Hu/E6), AF174305 (DS-1/Hu/E2), EU636933 (RRV/Si/E3), EF554157 (OVR762/Ov/E2), AF093199 (Wa/Hu/E1), AF165066 (I321/Hu/E2), FJ347131 (Guanaco/Arg/Rio Negro/1998/E12), FJ347109 (Guanaco/Arg/Chubut/1999/E12), FJ422140 (PTRV/Si/E2), EU839975 (SK277/Hu/E1), DQ146680 (Matlab13–03/Hu/E1), EF554091(B1711/Hu/E2), AY050273 (WC3/Bo/E2), EU753968 (mcs/13–07/Hu/E1), and D89873 (AU-1/Hu/E3).

NSP5

EF672583 (DS-1/Hu/H2), AY787651 (TB-chen/Hu/H2), DQ838630 (SA11-H96/Si/H5), AB008656 (AU-1/Hu/H3), DQ205231 (30/96/Lp/H3), AB091726 (Wa/Hu/H1), DQ494400

(KJ75/Bo/H1), DQ916134 (CMP034/Po/H1), EF672618 (ST-3/Hu/H1), DQ146670 (Dhaka12–03/Hu/H1), FJ169863 (02V0002G3/Av/H7), FJ347132 (Guanaco/Arg/Rio Negro/1998/H3), FJ347110 (Guanaco/Arg/Chubut/1999/H3), FJ422141 (PTRV/Si/H3), EU839976 (SK277/Hu/H1), DQ146681 (Matlab13–03/Hu/H1), DQ490561 (RV176–00/Hu/H2), EF554092 (B1711/Hu/H2), EF990702 (WC3/Bo/H3), EU753967 (mcs/13–07/Hu/H1), and AB009628 (PO-13/Av/H4).
